# S14-3: The adoption and implementation of a physical activity referral pathway integrated into a major U.S. health system

**DOI:** 10.1093/eurpub/ckae114.262

**Published:** 2024-09-26

**Authors:** Mark Stoutenberg

**Affiliations:** Durham University

## Abstract

**Background:**

Regular physical activity (PA) is highly effective in improving mental, physical, and emotional health; yet, health systems experience challenges integrating PA referral pathways connecting eligible patients to health-enhancing PA resources and programs.

**Purpose:**

To examine the adoption of a clinic-to-community PA referral pathway in primary care clinics in a major U.S. health system and identify provider- and patient-level barriers and facilitators associated with its implementation.

**Methods:**

Exercise is Medicine Greenville® (EIMG®) is a clinic-to-community model that connects primary care patients to a 12-week, evidence-informed, PA program in community PA facilities. Resumption of EIMG® in March 2021 after COVID-19 closures provided a unique opportunity to comprehensively evaluate the PA referral pathway from inception. Referral provision, utilization, and program enrollment were tracked from reopening through September 2022 in twelve primary care clinics. Semi-structured interviews were conducted with providers (informed by the i-PARIHS framework) and referred patients who declined enrollment (informed by the COM-B model). Interviews were recorded, transcribed, and themes identified.

**Results:**

Twenty-six providers completed interviews. Referral barriers included a lack of knowledge about EIMG®, a perceived time-consuming referral process, and placing incomplete referrals. Facilitators included acknowledgment of a high value for PA in healthcare, desire to incorporate EIMG® into patient visits, and the work of the EIMG® nurse navigator. Twenty-eight patients completed interviews. Enrollment barriers included time, financial constraints, inconvenient locations, disruptions due to COVID-19, and suboptimal communication. Facilitators included aligning EIMG® with patients’ health goals, enhanced trust due to provider referral, group-based format of the PA program, and friendly staff.

**Conclusions:**

Improving communication, conducting refresher trainings, simplifying the referral process, and providing more resources (for providers), along with providing more program details, and offering more program times, locations, and financial support (for patients), may increase success of PA referral pathways implemented in primary care settings.

**Practical implications:**

This work identifies modifiable factors that can guide improvements in the adoption and implementation of PA referral models in health settings.

**Funding:**

The Duke Endowment and the National Heart, Lung, and Blood Institute (NHLBI)

